# An educational intervention to prevent overweight in pre-school years: a cluster randomised trial with a focus on disadvantaged families

**DOI:** 10.1186/s12889-019-7595-2

**Published:** 2019-11-01

**Authors:** Alison Hodgkinson, Janice Abbott, Margaret A. Hurley, Nicola Lowe, Pamela Qualter

**Affiliations:** 1 School of Psychology, University of Central Lancashire, Preston, Lancashire PR1 2HE UK; 2 Faculty of Health and Wellbeing, University of Central Lancashire, Preston, Lancashire PR1 2HE UK; 3 School of Sport and Health Sciences, University of Central Lancashire, Preston, Lancashire PR1 2HE UK; 4 Institute of Education, University of Manchester, Manchester, M13 9PL UK

**Keywords:** Obesity, Early years’ centres, Healthy heroes, Cluster randomised trial, Cluster randomized trial, Parenting

## Abstract

**Background:**

Early prevention is a promising strategy for reducing obesity in childhood, and Early Years settings are ideal venues for interventions. This work evaluated an educational intervention with the primary aim of preventing overweight and obesity in pre-school children.

**Methods:**

A pragmatic, cluster randomised trial with a parallel, matched-pair design was undertaken. Interventions were targeted at both the cluster (Early Years’ Centres, matched by geographical area) and individual participant level (families: mother and 2-year old child). At the cluster level, a staff training intervention used the educational resource *Be Active, Eat Healthy.* Policies and provision for healthy eating and physical activity were evaluated at baseline and 12-months. The intervention at participant level was the *Healthy Heroes Activity Pack*: delivered over 6 months by Centre staff to promote healthy eating and physical activity in a fun, interactive way. Child and parent height and weight were measured at four time-points over 2 years. The trial primary outcome was the change in BMI z-score of the child between ages 2 and 4 years. Secondary outcomes consisted of parent-reported measures administered at baseline and two-year follow-up.

**Results:**

Five pairs of Early Years’ Centres were recruited. Four pairs were analysed as one Centre withdrew (47 intervention families; 34 control families). At the cluster level, improvement in Centre policies and practices was similar for both groups (*p* = 0.830). At the participant level, the intervention group reduced their mean BMI z-score between age 2 and 4 years (*p* = 0.002; change difference 0.49; 95% CI 0.17 to 0.80) whereas the control group showed increasing BMI z-score throughout. Changes in parent-reported outcomes and parent BMI (*p* = 0.582) were similar in both groups.

**Conclusions:**

The *Healthy Heroes* educational resource deterred excess weight gain in pre-school children from poor socioeconomic areas. With training, Early Years’ staff can implement the *Healthy Heroes* programme.

**Trial registration:**

ISRCTN22620137 Registered 21st December 2016.

## Introduction

The rising incidence of obesity and in particular childhood obesity is a major public health concern worldwide [1, 2]. In 2016, 11% of 2 to 4-year olds, more than 20% of 4 to 5-year olds and one-third of 11-year olds were overweight or obese in England [3, 4]. Childhood obesity is not only associated with a greater chance of premature death and disability in adulthood [5, 6] but obese children may experience health and psychological problems in childhood [7, 8] including cardiovascular dysfunction [9], type 2 diabetes [10], asthma [11], obstructive sleep apnea [12], bullying, poor body image and depression [13–15].

Numerous campaigns have attempted to reduce obesity with minimal long-term effects. This may be, in part, because obesity is already established, suggesting that preventative strategies are essential [16]. Many parental, societal and behavioral factors contribute to the causes of obesity [17–22]. Low levels of physical activity are associated with the development of excessive fatness in children and adolescents [23]. Parents’ eating behaviours influence their children’s eating patterns and weight development [24], and children’s eating behaviours and parental feeding patterns differ between underweight, normal weight and overweight children [25]. Improving parental knowledge, children’s diet and physical activity levels are important aspects for preventing obesity early in life and a valuable target for obesity prevention in young children is the childcare setting [26–28]. Systematic reviews have evaluated interventions with children aged 3 to 6 years [29–33], but only a few interventions worldwide have targeted younger children in childcare settings. With professional training, pre-school providers were able to implement child obesity prevention practices effectively [28, 34].

The prevalence of child obesity is associated with socioeconomic status [35]. Compared with children in higher socioeconomic groups those in lower groups have more than twice the prevalence of obesity and are less likely to consume five fruit and vegetables per day [2]. For children with obese parents, the increase in adiposity is also greater in lower socioeconomic groups [18]. There is an urgent need to reduce socioeconomic disparities. Early prevention is considered the most promising strategy for reducing obesity in childhood [36] and Early Years settings are ideal venues for obesity prevention interventions [37, 38]. Indeed, the core purpose of the Sure Start Early Years’ Centres in the UK, launched in 1999, was to improve health outcomes for young children (0 to 4 years) and their families (with a focus on the 20% most disadvantaged families) by providing a variety of services. This included advice and support to enhance parenting aspirations, self-esteem and parenting skills, together with child and family health and life chances (https://www.foundationyears.org.uk/childrens-centres/). With the opportunity to provide interventions at two levels: (a) with pre-school ‘Sure Start’ child care providers and (b) with families (parent and 2-year old child) a cluster randomised trial (CRT) was undertaken, with the primary aim to prevent excess weight gain measured as BMI z score at two-year follow-up.

## Methods

### Design, cluster recruitment and randomisation

The study was a pragmatic, cluster randomised trial (CRT) with a parallel, matched-pair design. The clusters were Sure Start Early Years’ Centres, matched in pairs by disadvantaged geographical area (populations in the pairs shared similar demographics). Inclusion criteria were (1) located in one of the 12 district authorities of Lancashire, UK, (2) located in an area of deprivation, (3) high levels of overweight/obese reception class children (4 to 5 years) in the neighbouring primary school, (4) not previously taken part in the intervention and (5) has a matched Centre in the geographical locality. Centres were excluded if they had previously accessed any part of the intervention being evaluated. The matched Centres were randomly allocated as intervention or control Centres simply by picking their name from a hat. Ten Centres were recruited in five matched pairs and all Centres had agreed to be allocated to either the intervention or control arm of the study. Blinding of the intervention was not possible as Centres needed to be aware of their intervention/control allocation given that staff in the intervention Centres had to be trained to deliver the intervention and families were required to be actively engaged in the intervention.

### Recruitment of families

Each of the ten Centres had around 800 children, with approximately 160 2-year olds per Centre registered on their databases. Early Years’ Centres sent out recruitment letters to 160 (10%) parents with a 2-year old child on their registers. Only one parent responded. Therefore, with the assistance of the Centre staff, one of the authors spent time at each Centre, recruiting parents with a 2-year old child into the study. Ethical approval was obtained from the University of Central Lancashire Ethics Committee. Written consent was obtained from each Centre manager. Parents provided written consent for themselves and consent on behalf of their child. Children also consented verbally.

### Interventions

**Fig. 1:**
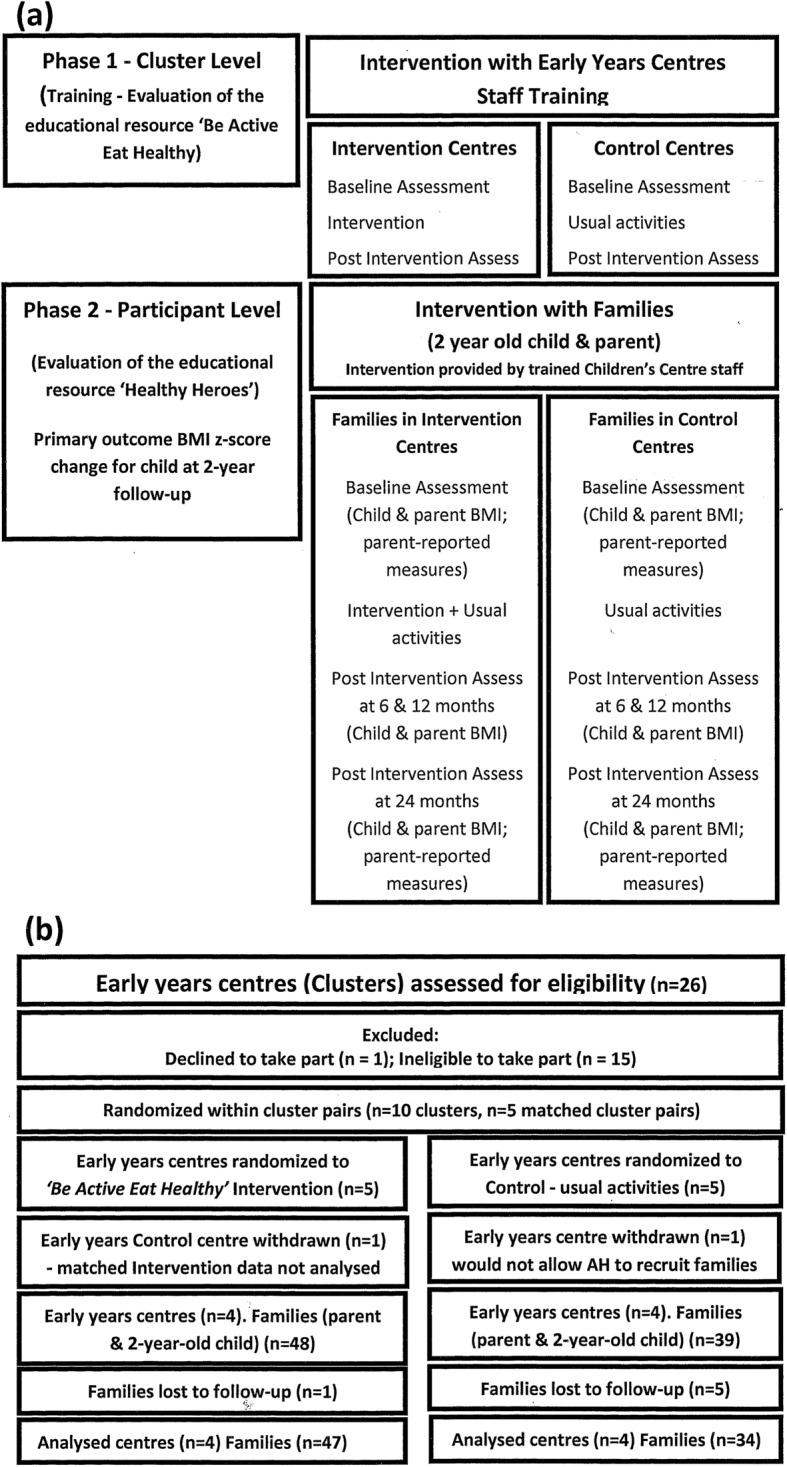
(**a**) Study design and intervention (**b**) Flow of clusters (Early Years Centres) and families from recruitment to analysis

Interventions were targeted at both the cluster (Early Years’ Centres) and individual (families) participant level (Fig. 1a). The educational health promotion resource *Be Active Eat Healthy* was an overarching initiative developed by a multi-agency team aimed at Early Years settings to help improve their policy on food and drink. The key element of the resource was a curriculum pack called *Healthy Heroes*. Subsequently, Early Years’ staff were trained to deliver *Healthy Heroes* to educate families about eating healthy and being active [39, 40] (www.lhsp.org.uk/healthyheroes).

#### Intervention at Centre level (staff training)

Training for staff (two-hour session) was provided by Lancashire County Council’s Children and Young People’s Team, NHS Public Health personnel and NHS Early Years Health Practitioners, in each Children’s Centre, using *Be Active, Eat Healthy* [39]. Two or three staff were trained at each Centre including a designated person who led the implementation of the Resource. Additionally, one or two staff members from each centre were trained to deliver group or one-to-one sessions with parents and their children: 1-day training on food preparation and healthy eating and 1-day training on physical activity, practice and provision. Training focused on Children’s Centre (a) policies: ‘Food and Drinks’ and ‘Physical Activity’, (b) provisions: snacks/meals served, cooking, food growing and active play, (c) practice: breastfeeding, weight and (d) health promotion: displays, leaflets, advice on nutrition and physical activity.

#### Intervention at family level (healthy heroes)

The intervention at family level was the *Healthy Heroes Activity Pack* developed by the Healthy Schools Team in Lancashire, UK, for primary school children. With theoretical underpinnings from Social Learning Theory, it aimed to promote healthy eating and physical activity in children and their families in a fun and interactive way. Children, parents and teachers rated it highly and anecdotal data suggested that families were making behavioural changes [40]. Subsequently, an Early Years’ version of the resource was developed for 2-year to 5-year olds in which the Healthy Heroes are four brightly coloured characters who do healthy activities advised by Freddy the Frog; for example, ‘eat breakfast’ and ‘go to the park’. Trained children’s Centre staff delivered the resource over a period of 6 months after baseline data collection. They used 24 activity cards (e.g. eating at the table), stickers, a song, puppet and utilised the Change4Life materials [41] (national campaign aimed at improving children’s diet/activity levels) which includes Start4Life. They delivered healthy cookery courses, active play sessions and gave the activities cards to families to use at home. This delivery was done in groups and one-to-one family support sessions in existing timetabled sessions and in specific *Healthy Heroes* sessions. The intervention was delivered to 81 families comprising a mother and her 2-year old child; 162 participants in total. All caregivers recruited were mothers since very few other related caregivers attended the Early Years Centres.

A baseline interview was arranged for each family at their Sure Start Centre. Demographic information was obtained, parent-report questionnaires administered and parent and child were weighed and their height measured**.** At 6, 12 and 24-months post-baseline, visits to the family home of intervention and control families enabled child and parent weight and height measurements. At 24-months the parent-reported questionnaires were also administered again. Each family was given a family leisure voucher for use at their local facilities and thanked for taking part.

### Outcome measures

#### Centre level (staff training)

In order to assess policies, provision and health promotion on healthy eating and physical activity in the Early Years’ Centres a Children’s Centre Assessment Tool (CCAT) was developed by a multi-disciplinary group of experts using National Institute of Clinical Excellence guidance and subject knowledge [40]. Baseline assessments (undertaken pre-randomisation of Centres) and 12-month follow-up assessments with Children’s Centre Managers and a member of staff were carried out face-to-face and employed a structured interview using the 32-item CCAT (Table 1). Four themes were explored: staff training (6 items), curriculum (4 items), policy (7 items) and practice related to food/nutrition and physical activity (15 items). Staff were asked to provide training records and evidence of programmes/plans. Each CCAT item was scored: 0 = not achieved, 1 = partially achieved, 2 = fully achieved. Theme scores were converted to percentage values to enable comparison between the four themes.

**Table 1:** Children’s Centre Assessment Tool (CCAT)

1. An identified member of staff to oversee food/drink	
2. Training for the workforce in Nutrition	
3. Training for the workforce in Food hygiene	
4. Training for the workforce in Practical Food Skills	
5. Training for the workforce in Oral Health	
6. Training for the workforce in Breastfeeding	
7. Healthy Eating Policy	
8. Uses local data and supports national/local priorities	
9. Signed up and promotes change4life	
10. Supports the uptake and continuation of breastfeeding	
11. Good practice in oral health promotion	
12. Involves children and parents in planning and delivery	
13. Provides a welcoming eating environment	
14. Availability of healthy food and snacks	
15. Opportunities to learn about food and a balanced diet	
16. Benefits of fruit and veg, limiting salt and sugar	
17. Provide cooking opportunities	
18. Hold discussion about food likes and dislikes	
19. Learn where food comes from	
20. Learn about good oral health	
21. Involves external professions to support healthy eating	
22. Weight Management advice and signposting	
23. Support staff to eat healthily	
24. Promote, provide and evaluate physical activity	
25. Provide daily opportunities for physical activity	
26. Actively promotes the 60 min a day message	
27. Involves children in planning and identifying barriers	
28. Uses community resources to deliver physical activity	
29. Promotes walking	
30. Encourage parents to take part in planning and delivery	
31. Provides physical activity training for staff	
32. Encourage staff to be active	

Staff were asked to provide training records and evidence of programs/plans. Each CCAT item was scored: 0 = not achieved, 1 = partially achieved, 2 = fully achieved

#### Family level (healthy heroes)

##### Demographic measures

To characterise the sample, a comprehensive profile of demographic variables was obtained for the child and parent (Table 2). Socioeconomic status was categorised by the highest parental occupational status according to the Office for National Statistics Standard Occupational Classification [42]. There are five categories of occupation: 1 = Professional, 2 = Managerial/Technical, 3 = Skilled, 4 = Partially skilled and 5 = Unskilled. These can be collapsed into professional (‘white collar workers’, categories 1 and 2) and non-professional (‘blue collar workers’, categories3, 4 and 5) as presented in Table 2.

**Table 2:** Baseline demographic characteristics of participants in the study

	Intervention (*n* = 47)		Controls (*n* = 34)	
	Mean	(SD)	Mean	(SD)
CHILD				
Height (cm)	88.0	(4.9)	90.5	(4.3)
Weight (kg)	13.2	(2.2)	13.2	(1.5)
	N	(%)	N	(%)
Age (months)				
24/25	24	(51)	15	(44)
26/27	7	(15)	3	(9)
28/29	5	(11)	4	(12)
30+	11	(23)	12	(35)
Gender				
Male	22	(47)	19	(56)
BMI centile				
1st to 90th	31	(66)	31	(91)
91st to 97th	7	(15)	2	(6)
98th +	9	(19)	1	(3)
Family visits to Centre (per week)				
1–2	32	(68)	7	(21)
3–4	11	(23)	21	(61)
5+	4	(9)	6	(18)
PARENT				
BMI	26.5	(5.8)	25.4	(4.8)
	N	(%)	N	(%)
Age (years)				
19–29	19	(40)	16	(47)
30–34	17	(36)	9	(27)
35–41	11	(23)	9	(27)
Gender				
Male	1	(2)	0	(0)
Ethnicity				
Asian	6	(13)	4	(12)
Black	0	(0)	2	(6)
White	41	(87)	26	(76)
Other	0	(0)	2	(6)
Paid employment				
None	24	(51)	16	(47)
Part-time	19	(40)	15	(44)
Full-time	4	(9)	3	(9)
Socioeconomic group				
Professional/managerial/technical	6	(13)	6	(18)
Skilled/partially-skilled/unskilled	41	(87)	28	(82)
Marital status				
Single	8	(17)	8	(24)
Partnered	13	(28)	12	(35)
Married	25	(53)	14	(41)
Divorced	1	(2)	0	(0)

##### Anthropometric measures

The parent and child were weighed without shoes in light clothing using calibrated Salter Electronic scales, measured in kg to the nearest 0.1 kg. Height was measured in centimetres, to the nearest millimetre, with a Seca Leicester Height Measure. All measurements were taken by one researcher and the NHS National Child Measurement protocol was followed [43].

#### Primary outcome measure

The primary outcome for the cluster trial of the *Healthy Heroes* Education resource versus control was the change in BMI z-score of the child participant between ages 2-years and 4-years. The BMI z-score was calculated using World Health Organisation standards [44] and the change was calculated as BMI z-score at age 4-years minus the BMI z-score at age 2-years.

### Parent-report questionnaire measures

#### Child eating behaviour questionnaire

The Child Eating Behaviour Questionnaire (CEBQ) is a 35-item instrument, validated for use with children as young as 2-years [45]. It has 8 scales: food responsiveness, enjoyment of food, emotional over-eating, desire to drink, satiety responsiveness, slowness in eating, emotional under-eating and fussiness. Internal reliability coefficients ranged from .74 to .91 and test-retest reliability ranged from *r* = .52 to *r* = .87. Parents rated the frequency of their child’s behaviour on a 5-point scale (1 = never to 5 = always) [45].

#### Parental feeding style questionnaire

The Parental Feeding Style Questionnaire (PFSQ) incorporates 27-items within four scales: emotional feeding, instrumental feeding, encouragement to eat and control over-eating. Cronbach alpha coefficients ranged from .67 to .83 and test-retest reliability ranged from .76 to .83. Each item had a 5-point Likert-scale [46].

#### Food choice questionnaire

The 36-item Food Choice Questionnaire (FCQ) has nine subscales: health, mood, convenience, sensory appeal, natural content, price, weight control, familiarity and ethical concern [47]. Each item has a 4-point Likert-scale (not important = 1 to very important = 4). The measure has good internal reliability (Cronbach alpha = .72 to .86) and test-retest reliability (.71 to .83) [47].

#### Warwick-Edinburgh mental well-being scale

The Warwick-Edinburgh Mental Well-being Scale (WEMWBS) is a single scale of 14 items, with five response categories [48]. Internal reliability (Cronbach alpha = .91) and test-retest reliability (Intraclass correlation coefficient = .83) are good. Items scores are summed to give a total score ranging from 14 to 70, with higher scores representing higher levels of mental well-being. Parents rated their own level of well-being. From a sample of 7020 in England, a population mean/norm of 51.6 was established [49].

### Sample size determination

With CRTs, standard approaches to sample size determination cannot be readily applied [50]. Additionally, there was an absence of studies in very young children with weight/BMI data to inform sample size estimates, in particular, intraclass correlation was unknown. Therefore, the sample size for the study was determined by practical considerations.

### Statistical analyses

Baseline characteristics in the intervention and control groups were compared using summary statistics. Provided the total CCAT scores for the Centres showed no significant deviation from normality, a paired t-test assessed CCAT score change within each group and an unpaired samples t-test compared change between groups.

The statistical significance of the difference in change in BMI z-score between the groups was tested using a number of related tests to ensure robust conclusions. In general, a 5% significance level was used to judge statistical significance. Testing at the Centre level was by a paired and an unpaired t-test using the centre means. The paired t-test used the pairing of the centres by socioeconomic profile and respected the original study design. The unpaired t-test ignored the pairing; if the correlation between pairs was small then the unpaired t-test has greater power. Subsequently, the extent of intraclass correlation (ICC) was assessed using a one-way ANOVA using the minimum and maximum number of children per Centre. Although the number of Centres was small, the Centre means were based on at least 8 children per Centre and so were approximately normal and use of t-tests and ANOVA was justified. Further, BMI z-scores would be normally distributed since this is used in their derivation. At the cluster level, tests of normality would have low power and so were not undertaken. Thirdly, the clustering within Centres was ignored and change in BMI z-score was tested using an unpaired t-test for the difference in BMI z-score change between the two groups. This assumed that the intraclass correlation was zero. The sample sizes in both the intervention and control groups exceeded 30 children and so means were normal to a good approximation. Finally, two multilevel models were fitted, allowing the variance between Centres and between children within Centres to be jointly estimated together with the effect of the intervention versus control, and thus allowed for non-ignorable intraclass correlation. The first model used BMI z-score change as the outcome, the second used BMI z-score at 4-years as the outcome but included BMI z-score at 2-years as a covariate. The second was used to provide robustly for the case where the baseline mean z-score was different between the intervention and control children.

For the parent-reported measures, a paired t-test was used to indicate whether the post-intervention measures were significantly different to pre-intervention measures for the groups separately. Normality was justified since the samples exceeded 30 in both groups. *P* values from the tests were used to identify measures which had changed in one group, but had not changed in the other group. Further, an independent sample t-test tested whether change was significantly different between the two groups with adjustment for baseline value. *P* values were used as a guide to interpretation to avoid problems with multiple testing and to respect the American Statistical Association’s advice on their use [51].

## Results

Figure 1b provides a flow diagram of the progress of five matched pairs of Early Years Centres (clusters) and individual participants through phases of the trial, and those lost to follow-up, in accordance with the CONSORT statement for cluster randomised trials [52].

### Intervention at Centre level (staff training)

The overall improvement in Centre policies and practices from pre to post-intervention was greater in the intervention group (18%) compared to the control group (16%) although the difference was not significant (*p* = 0.83). However, the improvement in ‘training’ within the intervention Centres (37%) was significantly better than the Control centres (3%) (Table 3). Although not significant, it is notable that greater improvements were made in the control group regarding the ‘curriculum’ (10%) and ‘practice’ (9%) aspects of the assessment.

**Table 3:** Means (SD) of Centre total scores for each of the CCAT domains together with the Centre mean total score as a percentage of total domain score

	Intervention group *N* = 5			Control group *N* = 5			Change *p* Value
	Pre	Post	Change	Pre	Post	Change	
Training (range 0–12)	58% 7.00 (2.24)	95% 11.40 (0.89)	37% 4.40 (2.61)	52% 6.20 (2.49)	55% 6.60 (3.36)	3% 0.40 (1.34)	.028
Curriculum (range 0–8)	55% 4.40 (3.78)	60% 4.80 (2.28)	5% 0.40 (1.52)	65% 5.20 (0.45)	80% 6.40 (0.89)	15% 1.20 (0.84)	.343
Policy (range 0–14)	61% 8.60 (1.67)	84% 11.80 (0.45)	23% 3.20 (1.30)	37% 5.20 (1.79)	54% 7.60 (2.70)	17% 2.40 (3.36)	.641
Practice (range 0–30)	67% 20.0 (4.47)	78% 23.4 (3.36)	11% 3.40 (4.83)	45% 13.60 (2.70)	65% 19.60 (7.77)	20% 6.00 (6.08)	.478
Total (range 0–64)	62% 40.00 (10.68)	80% 51.40 (5.55)	18% 11.40 (9.07)	47% 30.20 (3.70)	63% 40.20 (13.44)	16% 10.00 (10.70)	.830

### Intervention at family level (*Healthy Heroes*)

Ten centres were recruited, but one pair of Centres was withdrawn because the control Centre refused to allow families to be recruited to the study.

#### Baseline characteristics of children and parents

The baseline characteristics of children and parents in the intervention and control groups are provided in Table 2. Overall, the children in the intervention and control groups were similar except in two aspects: the control group children made more Centre visits per week and the intervention group had proportionally more children above the 98th BMI percentile. The mothers in the intervention and control groups were similar except the control group had twice the proportion of smokers.

#### Primary outcome

**Fig. 2:**
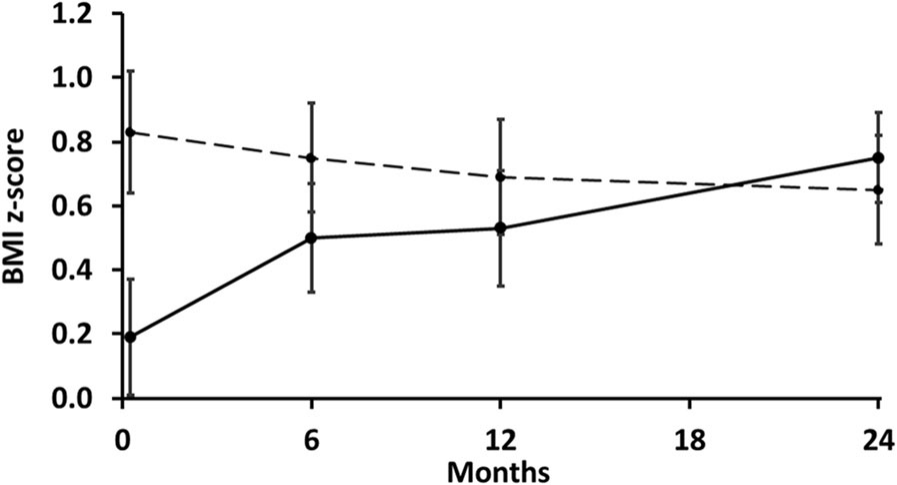
BMI z-scores means (●) for the Healthy Heroes educational intervention (dashed line) and the control (solid line), bars are plus and minus one standard error of the mean

The trial primary outcome for the intervention with families was the child BMI z-score. Mean values over the 2-year study are shown in Fig. 2. The intervention group reduced their mean (SE) BMI z-score between 2-years and 4-years of age whereas the control group showed increasing BMI z-score throughout the study period (Tables 4 and 5). At Centre level, both the paired (*p* < 0.043) and independent (*p* < 0.004) samples t-tests comparing BMI z-score change from 2-years to 4-years, providing strong evidence of an effect of the intervention compared to control (Tables 4 and 5). The unpaired analysis at the cluster level had greater power because the correlation between the matched pairs of Centres was found to be negligible. The presence of intraclass correlation did not invalidate the cluster level analysis. At the level of the individual child, when change in the BMI z-score was fitted as the outcome in a multilevel model, there was, again, strong evidence of an effect of the intervention regardless of whether intraclass correlation was assumed zero or whether it was estimated (*p* < 0.001). However, children in the intervention group had on average higher BMI z-score at the start of the study compared to the children in the control group. To adjust for that, the multilevel model was fitted again, with BMI final z-score as the outcome and initial BMI z-score included as a covariate. Again, the intervention was significant (*p* = 0.002). The difference in change was estimated at 0.49 (95% CI 0.17 to 0.80) indicating greater mean reduction in BMI z-score for the children in the intervention group compared to the children in the control group (Tables 4 and 5) for the same initial BMI z-score. In effect the children belonging to the intervention group reduced their mean BMI z-score whereas those in the Control group increased their mean BMI z-score with a mean difference of 0.49 BMI z-score units.

**Table 4:** Baseline and final BMI z-scores of the study children and their parents

	n	Child age 2-years			Child age 4-years			Change		
		Mean	SD	SE	Mean	SD	SE	Mean	SD	SE
Child BMI z-score:										
Control centres										
C1	8	0.08	0.52	0.18	0.75	0.47	0.17	0.67	0.31	0.11
C2	9	−0.04	1.02	0.34	0.64	1.02	0.34	0.68	0.33	0.11
C3	8	0.78	1.63	0.58	0.97	1.16	0.41	0.19	0.92	0.33
C4	9	−0.00	1.03	0.34	0.67	0.65	0.22	0.67	0.62	0.21
All	34	0.19	1.11	0.19	0.75	0.84	0.14	0.56	0.60	0.10
Intervention centres										
H1	13	1.00	1.29	0.36	0.61	1.46	0.41	−0.39	0.69	0.19
H2	13	0.52	1.32	0.37	0.26	0.87	0.24	−0.26	1.04	0.29
H3	11	1.22	1.24	0.37	1.30	0.82	0.25	0.08	1.03	0.31
H4	9	0.57	1.50	0.50	0.47	1.07	0.36	−0.10	1.29	0.43
All	46^1^	0.83	1.32	0.19	0.65	1.13	0.17	−0.18	0.99	0.15
Parent BMI:										
Control centres										
All	34	25.35	4.83	0.83	25.83	5.09	0.87	0.48	1.36	0.23
Intervention centres										
All	47	26.53	5.81	0.85	27.28	6.64	0.97	0.75	2.94	0.43

^1^BMI z-score could not be calculated for one child due to morbid obesity beyond WHO range

**Table 5:** Statistical tests of change difference between control and intervention at the two-year follow-up

Intraclass correlation (ICC):	Min. n estimate		Max. n estimate		
Control	0.0396		0.0443		
Intervention	0.0000		0.0000		
	Estimate	SE	95% CI		*P* value
Analysis at the centre level:					
Outcome = final BMI z-score – baseline BMI z-score					
Control	0.55	0.12	0.16	0.94	
Intervention	−0.17	0.10	−0.49	0.16	
Change difference (centres paired)	0.72	0.21	0.04	1.40	0.043
Change difference (centres unpaired)	0.72	0.16	0.33	1.10	0.004
Analysis at the child level:					
Outcome = final BMI z-score – baseline BMI z-score, assuming ICC = 0					
Control	0.56	0.10	0.35	0.77	
Intervention	−0.18	0.15	−0.48	0.11	
Change difference (centres unpaired)	0.74	0.18	0.38	1.10	< 0.001
Outcome = final BMI z-score – baseline BMI z-score, assuming ICC > 0					
Centre level variance	0.00	0.00			
Child level variance	0.70	0.11			
Change difference (centres unpaired)	0.74	0.19	0.37	1.11	< 0.001
Outcome = final BMI z-score, baseline BMI z-score as covariate, assuming ICC > 0					
Centre level variance	0.00	0.00			
Child level variance	0.47	0.07			
Baseline BMI z-score	0.60	0.06			
Change difference (centres unpaired)	0.49	0.16	0.17	0.80	0.002

### Parent BMI

From children aged 2-years, parent BMI (Table 4) was not significantly different between the intervention and the control groups (*p* = 0.322); the change in parent BMI between ages 2-years and 4-years showed no significant difference between the groups (*p* = 0.582).

### Parent-report questionnaire measures

Parent-reported data are presented in Table 6. The only difference between the intervention and control groups which came close to statistical significance was for Emotional over-eating (*p* = 0.064) on the CEBQ, with an unexpected increase observed for the intervention group and a decrease for the control group. Apart from this, there were similar changes in both groups for many aspects of food/eating behaviour. An increase in Instrumental feeding on the PSFQ highlighted that, in both groups, mothers rewarded children with food for ‘good’ behaviours and punished them by removing food for ‘bad’ behaviours. Both groups attributed greater importance to Health, Convenience, Sensory Appeal and the Natural Content of food, on the FCQ post-intervention. It is notable that both intervention (46%) and control (45%) groups had mean values lower than the national average (52%) on the WEMWS, although parental well-being improved in both groups during the study.

**Table 6:** Parent-reported outcomes for intervention and control groups; mean (SD)

	Intervention group (*n* = 47)			Control group (*n* = 34)			
	Child 2-years	Child 4-years	*p* value^1^	Child 2-years	Child 4-years	*p* value^1^	*p* value^2^
*Child Eating Behaviour Questionnaire*							
Slowness in eating	2.6 (1.0)	2.9 (1.0)	0.055	2.8 (1.2)	2.9 (1.2)	0.654	0.554
Satiety responsiveness	3.0 (0.9)	3.2 (1.0)	0.040*	2.8 (1.1)	3.1 (1.0)	0.276	0.411
Food fussiness	2.7 (0.8)	2.8 (1.1)	0.488	2.8 (0.9)	2.8 (1.1)	0.562	0.996
Food responsiveness	3.0 (1.2)	3.2 (1.2)	0.104	3.5 (1.3)	3.2 (1.2)	0.174	0.118
Enjoyment of food	4.2 (0.9)	4.1 (1.0)	0.593	4.3 (1.1)	4.3 (1.0)	0.895	0.626
Desire to drink	3.2 (1.4)	2.9 (1.4)	0.394	3.2 (1.5)	2.7 (1.4)	0.029*	0.362
Emotional under-eating	3.7 (1.1)	3.8 (1.1)	0.480	3.5 (1.2)	3.7 (1.4)	0.379	0.885
Emotional over-eating	1.6 (0.7)	1.7 (0.7)	0.241	1.6 (0.6)	1.5 (0.6)	0.173	0.064
*Parental Feeding Style Questionnaire*							
Instrumental	2.7 (1.2)	3.3 (1.1)	0.002*	3.0 (1.3)	3.5 (1.2)	0.032*	0.802
Control	3.8 (0.8)	3.8 (0.7)	0.801	3.8 (0.8)	3.6 (0.9)	0.305	0.342
Emotion	1.9 (0.9)	1.9 (0.8)	0.568	2.2 (1.2)	1.9 (1.2)	0.052	0.124
Encouragement	4.5 (0.5)	4.7 (0.4)	0.019*	4.5 (0.5)	4.6 (0.5)	0.247	0.324
*Food Choice Questionnaire*							
Health	2.8 (0.7)	3.0 (0.6)	0.008	2.7 (0.8)	2.9 (0.7)	0.006	0.801
Mood	2.2 (0.7)	2.2 (0.7)	0.770	2.2 (0.7)	2.1 (0.7)	0.307	0.299
Convenience	2.6 (0.6)	2.8 (0.6)	0.001	2.7 (0.7)	2.9 (0.7)	0.017	0.747
Sensory Appeal	3.1 (0.4)	3.2 (0.5)	0.020	3.2 (0.6)	3.4 (0.5)	0.063	0.534
Natural Content	2.6 (0.9)	2.7 (0.8)	0.058	2.3 (0.9)	2.7 (0.9)	0.001	0.260
Price	2.7 (0.7)	2.7 (0.8)	0.935	2.9 (0.8)	3.0 (0.8)	0.475	0.426
Weight Control	2.3 (0.9)	2.5 (0.9)	0.010	2.3 (0.8)	2.4 (0.9)	0.414	0.320
Familiarity	2.7 (0.8)	2.8 (0.6)	0.152	2.5 (0.8)	2.6 (0.7)	0.187	0.594
Ethical concern	1.8 (0.9)	1.8 (0.8)	1.000	1.5 (0.7)	1.6 (0.7)	0.571	0.740
*Warwick-Edinburgh Mental Well-being Scale*							
Total score	45.9 (9.2)	50.6 (10.7)	0.002	45.0 (9.9)	48.6 (11.3)	0.056	0.633

^1^ *P* value for test of zero change (post-pre) within group

^2^*P* value for test of difference in change between intervention and control groups, adjusting for difference at baseline using baseline value as a covariate

^*^p value less than 0.05

## Discussion

The trial primary outcome (change in BMI z-score of the child participant between ages 2-years and 4-years) indicated that the study had prevented gain in overweight/obesity in the *Healthy Heroes* intervention group at two-year follow-up. The intervention group had a higher baseline BMI z-score compared to the control group. This lack of balance is common in cluster trials where randomisation is not at the individual level but at the cluster level and is likely a result of recruitment bias by Children Centre staff, subconsciously selecting those they thought could benefit most. Therefore, some of the observed effect of the intervention was due to regression to the mean but the final analysis using baseline BMI z-score as a covariate in a multilevel model adjusted for this. This reduced the effect size from three quarters of a z-score unit in the less complex analyses to about half a z-score unit in the final analysis.

The *Healthy Heroes* intervention was effective in terms of BMI z-score with a difference in change of BMI z-score units of 0.49 in favour of the intervention. Categorically, this equates to the percentage of children classed as overweight/obese pre to post-intervention decreasing from 34 to 21% in the intervention group and increasing from 9 to 21% in the control group. However, the reason/s for this are unclear given the cluster level data regarding staff training and the parent-reported outcome data. An overall improvement in Early Years Centre policies and practices was seen in both intervention and control Centres. Just being assessed was sufficient to encourage control Centre staff to implement some changes. Indeed, the control Centres made greater improvements in aspects of the ‘curriculum’ and ‘practice’, delivering healthy eating and exercise messages to families and provided them with encouragement. Hence, a monitoring-only implementation approach was just as effective as a training and monitoring implementation approach. As some Centres were at ceiling on the CCAT at baseline (fully implementing a specific practice or having a specific policy in place), theme change values alone are not informative. The sensitivity of the CCAT may be improved with more than three response options. On an item level, compared with control Centres, intervention Centres ‘promoted, provided, and evaluated physical activity’ such as walking, provided physical activity training for staff and involved external professions to support healthy eating. Those behaviours may have contributed to the changes in child BMI. Professional training appears to be important given that the personal beliefs and practice of staff with regard to food, nutrition and physical activity influence their practice with children [53]. Training for Children’s Centre staff is imperative as they are often low paid and poorly educated. However, informal discussions with families provided feedback that they had received the intervention as intended from the staff.

The parental reports on the CEBQ, PFSQ or FCQ cannot easily explain the changes in BMI. The only difference on the CEBQ between the intervention and control groups which came close to significance was for Emotional over-eating. This was not as expected as there was a decrease in the control group and an increase in the intervention group suggesting that children in the intervention group were more likely to eat in response to emotional cues. This cannot be easily explained. In line with other researchers [54], the factor structure of the CEBQ could not be replicated in this study, casting doubt on the value of analysing the data using the original subscales. Item analyses highlighted that children in the intervention group ate more slowly at two-year follow-up together with increased parental recognition that their child was asking for food more frequently. However, the CEBQ does not differentiate between the types of food and it is possible that children were asking for the foods that they had been learning about (e.g. fruit and vegetables) as part of the *Healthy Heroes* activities. It is also possible that children in the intervention group had increased their physical activity levels, making them feel hungry, which could potentially justify the increase in the amount of food they ate as well as the frequency they asked for food. The lack of parent-reported physical activity is a limitation of this work. The assessment of physical activity either by parental report or a monitoring device would enhance the interpretability of the data.

Both intervention and control groups reported more instrumental feeding on the PFSQ. Such food–reward behaviour is recognised as obesogenic as typically, energy-dense and nutrient-poor foods such as chocolate and sweets are given [55] and is independently associated with binge eating in adulthood [56]. This may simply reflect the age/stage of development of these children and the typical initiating of rewards and punishments by parents, or this pattern of parenting may be more common in areas of social/educational deprivation. It is not surprising that parental well-being was initially low in this sample. At two-year follow-up, both groups had scores that were similar to the national average. Again, this may be due to the age/developmental stage of the child, with children starting full-time education around the time of their final follow-up assessment or welcomed attention from being in the study. It is noteworthy that all the questionnaires proved challenging: many families had poor levels of literacy, requiring time-intensive, face-to-face interviews to complete them. How to assess meaningfully an intervention with hard to reach families in areas of social disadvantage requires new thinking. Engaging families in developing assessments is an appropriate way forward.

Successful pre-school interventions, in terms of BMI decrease/maintenance, have been reported by others [37, 38, 57–60]. Some had professional specialist trainers (e.g. child health professionals, nurses, social workers) delivering the interventions and working closely with pre-school Centre managers to write and deliver their nutrition and physical activity policies [38, 58, 61]. Other studies have observed a decrease in BMI in both control and intervention Centres [61, 62] suggesting that the attention given to the control Centres (evaluation of policy and practice) and families (evaluation of weight) may be sufficient to kick-start a change in attitudes and behaviour.

Conducting research on a ‘hard to reach’ population is challenging, evidenced by the fact that only 1 in 160 families responded to the initial invitation to take part. Cluster randomised trials are less common than traditional trials but they are ideally suited to interventions that need to be delivered to entire communities and can best capture the effects of intervening in such communities. They are also likely to prevent cross-contamination (where intervention families share health messages with control families in the same localities) [63]. Centres were not blinded as to whether they belonged to the intervention or control group. Group allocation concealment was not possible as staff in the intervention group were required to undergo training so that they could deliver the intervention and the families were asked to actively engage in healthy eating and physical activities. However, baseline Children’s Centre data were obtained from Centre staff regarding their healthy eating/activity practices, training, curriculum and policies prior to randomization. Additionally, Centres were allocated to clusters (as either intervention or control) before children/families were recruited. Potential staff bias could be prevented by recruiting families prior to group allocation but this can be difficult where intervention clusters need to receive training. The downside to a matched-pair design was the withdrawal of a control centre, which meant we had to exclude the matched intervention centre from the family level analyses. This resulted in the data of 81 families being analysed, with a greater number of intervention families than control families. This is not a problem in itself and is often inevitable with a cluster trial in which recruitment occurs after the randomisation of clusters but it is possible that the results from a larger sample may be different. However, a post hoc sample size calculation required 34 children per group. This study contained 34 children in the control group and 47 in the intervention group and had at least 80% power to detect the difference that it did indeed detect, namely a 0.49 difference (95% CI 0.17 to 0.80) in change of BMI z-score between the two groups. This was a small study but it demonstrates a proof of concept: namely that it is possible to intervene at an early age to prevent obesity before children start school. Future work should measure Centre staff training outcomes (knowledge, confidence, feasibility), parental engagement and the children’s involvement with the resource. The results of larger studies in which Early Years staff have been professionally trained and supported are awaited.

## Conclusions

This pragmatic intervention reached ‘hard to reach’ families in areas of social disadvantage. The *Healthy Heroes* educational intervention, provided to mothers and 2-year old children, was able to prevent excess weight gain at two-year follow-up when the children were 4 years of age. The percentage of children classed as overweight/obese decreased in the intervention group but increased in the control group. The behavioural mechanisms for this effect are unclear given that the parent-reported outcome data could not explain the changes in BMI and parental BMI was unchanged. Pre-school obesity interventions are feasible, and with training, Early Years’ staff can implement education programs.

## Data Availability

Data from the study are available from the corresponding author.

## Abbreviations

BMI: Body Mass Index
CCAT: Children’s Centre Assessment Tool
CEBQ: Child Eating Behaviour Questionnaire
CRT: Cluster Randomised Trial
FCQ: Food Choice Questionnaire
PFSQ: Parental Feeding Style Questionnaire
WEMWBS: Warwick-Edinburgh Mental Well-being Scale
